# Barriers to implementing problem-based learning at the school of medicine of Debre Berhan University, Ethiopia

**DOI:** 10.1186/s12909-024-05252-1

**Published:** 2024-05-06

**Authors:** Aklile Semu Tefera, Ermiyas Endewunet Melaku, Besufekad Mulugeta Urgie, Erzik Muhammed Hassen, Tilahun Deresse Tamene, Enguday Demeke Gebeyaw

**Affiliations:** 1 Department of Public Health, Debre Berhan University, Debre Berhan, Ethiopia; 2 Department of Internal Medicine, Debre Berhan University, Debre Berhan, Ethiopia; 3 Department of Surgery, Debre Berhan University, Debre Berhan, Ethiopia

**Keywords:** Problem-based learning, Qualitative study, Implementation barriers

## Abstract

**Background:**

Implementing PBL in teaching and learning can be challenging due to a variety of complex barriers. Studies on barriers to the implementation of problem-based learning in Ethiopia are scarce. This study aimed to explore the barriers to the implementation of problem-based learning at the Debre Berhan University Medical School.

**Methods:**

A qualitative study was conducted among faculty and medical students at the medical school. Purposive sampling was used to select participants. Semi-structured interviews were conducted with tutors and academic leaders, including the problem-based learning coordinator, the biomedical sciences coordinator, and the school dean. Data was also collected from students through focus group discussions. All interviews and discussions were recorded. The four steps of data analysis of Spradley, including domain analysis, taxonomic analysis, componential analysis, and theme analysis, were employed.

**Results:**

The study identified student-related, tutor-related, case scenario-related, and assessment-related barriers as the most significant obstacles to implementing problem-based learning. These barriers included work overload for both students and tutors, lack of training and experience among tutors, student reluctance, absence of standardized case scenarios, subjectivity of assessment methods, and on-the-spot assessment of students.

**Conclusions and recommendations:**

: Lack of both tutor and student commitment, lack of standardized cases, absence of a recognition of staff input, gap in communication skills, work overload, lack of continuous training, and at-spot evaluation of students were identified as the main barriers to the implementation of PBL.

## Introduction

Problem-based learning (PBL) is an instructional approach in which students work together in small groups, guided by a tutor, to solve problems and reflect on their experiences [1]. In this process, the learners, rather than the tutor, are active participants in the discussion, and each member of the group contributes to the learning. The PBL tutor is a facilitator and evaluator who provides feedback to the group. A case is usually discussed over two tutorials, each two hours duration. At the end of the first tutorial, the students identify learning goals that guide their self-directed learning [2, 3].

Compared to conventional curriculum, PBL is reported to have many advantages, including increased knowledge retention, improved problem-solving abilities, and better integration of basic science and clinical skills. Additionally, it has been assumed that the PBL approach promotes the utilization of social learning principles, which activates group discussion and therefore contributes to the development of interpersonal communication and presentation skills [4–6].

The evident benefits of PBL and the changing face of medicine and medical education have led many institutions to consider the adoption of PBL curricula [7].The introduction of PBL as part of the medical education curriculum has a relatively short history in Ethiopia. It was adopted as an educational strategy in the innovative medical education curriculum of 2011, which has been implemented in 13 medical schools of the country [8]. Debre Berhan University Medical College was one of those medical schools who adopted PBL as one of the key educational methods in 2011. Currently, it has been introduced in many other Ethiopian medical schools.

The program is designed for preclinical medical students. Teachers usually undergo two to three days of training before being assigned as tutors, although this training is not provided consistently. Each week, students engage in a problem-based learning (PBL) session based on a single case scenario, spanning two nonconsecutive days. On the first day, students receive the scenario of the case and study objectives and references, allowing them time to review the case. A detailed discussion will take place during the next session.

Despite its benefits, implementing PBL poses complex challenges in medical education. Research across developed and developing nations highlighted barriers including limited resources, questioning techniques, delayed facilitator responses, unawareness of individual learning goals, time demands for both teachers and students, large class sizes, information overload, low motivation, anxiety, lack of technology, and the unfamiliarity with PBL among traditional teachers [9–14].

A study addressing cracks in problem-based learning suggested focusing on training new staff, maintaining briefing/debriefing sessions, reviewing materials and program based on feedback, monitoring program delivery, reviewing management, aligning assessment with PBL principles, ongoing curriculum maintenance, providing ongoing tutor training, and resolving conflicts to maintain healthy PBL delivery [3].

Studies conducted on Barriers to Implementing Problem-Based Learning (PBL) in Sub-Saharan Africa, including Ethiopia, are limited. In Ethiopia, only limited research on PBL was published and was mainly focused on the knowledge and attitude of academics and students toward PBL. Therefore, this study was aimed to explore specific barriers to the implementation of problem-based learning (PBL) at Debre Berhan University, School of Medicine, that could serve as a trigger for further nationwide studies.

## Methods and materials

### Study design, settings and participants

A qualitative cross-sectional study was conducted at the Debre Berhan University medical school from February 1 to February 30, 2022. Debre Berhan University is located 130 km northeast of the capital Addis Ababa. The medical school consisted of 107 academic and 24 technical personnel during the study period. The school had a total of 117 year 1 and year 2 medical students during the study period.

### Sample size and sample procedures

In the student selection process, we chose students with diverse academic backgrounds, ensuring representation from various degree programs (first degree in health and health related departments was a requirement to join medical school). Additionally, we considered the academic performance of the previous semester to include students from different academic performance categories.

Consequently, we selected 16 students from year 1 and year 2(PBL is given only for year 1 and 2 students). These students were then divided into two groups, with eight students in each group from each year. We conducted group discussions and continued data collection until we reached saturation of ideas. Saturation was deemed reached when ideas became repetitive and the group facilitator concluded that no new insights were likely to emerge.

For staff selection, our focus was on academic staff members with substantial teaching experience in problem-based learning (PBL) and demonstrated active involvement in such instructional methods. Based on these criteria, we interviewed a total of 15 academic staff using a semi-structured questionnaire. Finally, academic leaders, including the biomedical sciences coordinator and the medical school dean, were interviewed.

### Data collection procedures

In the last week of January 2022, a pilot study was conducted to test the suitability and clarity of the interview guide. Constructive comments received from the pilot study participants were used to revise the interview guide. The data from the pilot study were not included in the results of the main study. Data collection for the main study was carried out by researchers from February 1, 2022, to February 30, 2022, using semi-structured interviews and key performance indicators (KPI).

By gathering data from these different sources, such as interviews and focused group discussions (FGD), a comprehensive understanding of the phenomenon and the achievement of the study objectives were ensured. This triangulation of data sources increased the credibility of the study findings. The data collected included information on sociodemographic, PBL cases, mode of delivery of PBL, and assessment of students.

The interview guide questions were initially prepared in English and then translated into Amharic language. An expert in both languages checked for consistency in the questions. Both the focus group discussion (FGD) and interviews were conducted and data was collected until saturation of ideas was reached, which occurred when ideas were repeatedly raised and the facilitator of the group (interviewer) believed that no new ideas would be raised, at which point the group discussion was stopped.

### Data quality management and confidentiality

To ensure data quality, all study participants were informed about the relevance of the study and the confidentiality of the information. The author ensured that data collection methods aligned with research objectives and are appropriate for the study. Pretest data collection was undertaken so that the validity and appropriateness of the questions included was questioned. Triangulation, using multiple sources of data, was used to improve the credibility and reliability of the findings. Saturation was also employed, in which data collection continued until data saturation was reached to ensure a comprehensive exploration of the research questions. The information collected from the patients was not used for any purpose other than the intended purpose mentioned in this research.

### Data analysis

Spradley’s four steps of data analysis [15], were utilized, including domain analysis, taxonomic analysis, component analysis, and theme analysis. In the first step, the data was examined to identify units of cultural knowledge that fell into larger categories based on their similarities or semantic relationships. This analysis was performed across the entire data set. The second step involved establishing a classification system for the identified domains, revealing the internal structure of each domain and the relationships among the categories within them. Moving on to the third step, the relationships between terms within each domain were identified and examined, as well as any differences among the terms or subcategories within a domain. Finally, in the fourth step, the researcher explored and uncovered the relationships among domains and themes throughout the entire data set. A theme map, consisting of a list of themes and subthemes, was developed and used to check the themes [16].

During this process, themes that did not generate meaningful data were discarded. The identified themes were revisited to identify similarities and differences in meaning. Furthermore, relevant literature from previous studies was reviewed to confirm the applicability of the identified themes and allow the researcher to draw the necessary inferences. As a result, four main themes and related subthemes were discovered, based on the characteristics and factors that influence the implementation barriers of Problem-Based Learning (PBL) within the School of Medicine.

## Result

### Sociodemographic characteristics of study participants

This study involved a total of 16 medical students, aged between 24 and 27, with a male majority of 12 participants. In parallel, 15 academic staff members participated in this study, and the majority of them [11] were male with a range of age from 30 to 35. All academic staff held the academic rank of lecturer, and both the school Dean and the biomedical science coordinator carry the designation of assistant professor.

### Theme 1: student-related barriers

#### Resistance of students towards PBL implementation

There were instances where students showed resistance to participating in Problem-Based Learning (PBL) sessions. One of the tutors said: “*Some students did not perceive PBL as an effective teaching methodology; they need us to tell them everything in detail*”. The tutor continued: “*This resistance is often observed in students who had previously experienced a more traditional teacher-directed approach to education during their high school and undergraduate courses at the university*”. Students encountered difficulties transitioning from a passive to an active learning environment and expressed fear of being challenged by tutor questions.

Although many students expressed interest and are comfortable teaching using Problem-Based Learning (PBL) methodology, they acknowledge that it requires significant time for preparation and self-study. Students said *‘we are asked to read a large volume of information in a short period of time; there is only a one-day gap between the two sessions of PBL and it is very difficult to cover all objectives of the session; we need to read all the basic sciences included in the session, which is very difficult and tidies’.* Unfortunately, some students attended PBL sessions without completing the necessary readings and preparations. Furthermore, there are students who believe that PBL sessions involve irrelevant discussions and consider them a waste of time.

#### Lack of consensus among students

In the context of Problem-Based Learning (PBL), students are required to collaborate within their teams. However, a common issue reported by many students is the absence of a shared understanding among team members. Some of the students reported that ‘some *of us lack the willingness to work towards the same goals and some group members do not show responsibility and some team members considered PBL useless and did not prepare’.* Students complained that these factors contribute to difficulties in effectively working together as a team.

#### Dominance of a few students in PBL sessions

One notable observation in problem-based learning (PBL) sessions is the tendency for a small group of students to dominate the discussions. One of the students said, ‘Some *students are overactive and wanted to take control of the conversation during the session.’ They wanted to tell everyone they know, even those issues that are not related to the session. And some of the tutors have the tendency to follow them instead of controlling them and giving chances to other students”.* Unfortunately, this dominance can hinder the participation of other students and limit their opportunities to contribute to the discussion.

#### High expectations of students towards tutors

Due to their unfamiliarity with this teaching methodology and the novelty of the curriculum, students do not fully understand their role as learners and the role of the tutor. Most of the tutors said: “*Some students expect much from us, they believe that we, the tutors, have the primary responsibility to discuss the whole issue during the discussion. They mistakenly believe that they are not required to invest significant effort and take ownership of their own learning process.'* These students often anticipate that their tutor will bear the primary responsibility for their learning, placing a heavy reliance on their guidance and support.

#### Insufficient communication skills hinder participation

Some students have been observed to struggle with shyness or discomfort when speaking in front of a group. One of the group members said that *‘we have a discomfort speaking in front of our friends and tutors; we also have language barriers to discuss our readings’. These* students found it challenging to express their thoughts and ideas confidently, leading to a reluctance to actively participate in discussions. In addition, language barriers pose another obstacle for some students. Those who are not fluent in the language of instruction encountered difficulties in articulating their ideas effectively.

### Theme 2: barriers related to tutors

#### Lack of motivation in the implementation of PBL

One of the significant barriers identified in the implementation of Problem-Based Learning (PBL) was the lack of motivation and unwillingness among tutors to deliver PBL sessions. Tutors have expressed three main reasons for their lack of motivation and resistance to participating in PBL.

First, the tutors have mentioned their dissatisfaction with their passive involvement during the sessions. *‘We feel that our time is wasted without making significant contributions to group discussions. This lack of active engagement diminishes our motivation to fully participate in the PBL process”.*

Second, the tutors have highlighted the challenge of addressing multidisciplinary subjects within the PBL framework. They said that “despite the fact that *the case requires knowledge from various disciplines, only one tutor, who is an expert in a single subject, is expected to lead the session. We find it tedious and tiresome to constantly read and prepare for subjects outside of our area of expertise, further reducing our motivation”.*

Lastly, tutors have expressed a lack of incentives for their participation in PBL. *‘There is no special recognition or reward for our efforts in facilitating PBL sessions, which diminishes our motivation to actively participate’.*

#### Work overload in PBL implementation

Another significant challenge that has been raised by both tutors and higher-level authorities, such as the department head and the school dean, is the issue of work overload. This concern stems from a shortage of staff available to serve as PBL tutors. The head of the biomedical department said that “*Currently, only a limited number of general practitioners and biomedical science instructors have received training to serve as a tutor and are responsible for delivering PBL sessions on a weekly basis’.* This shortage of trained tutors created a heavy workload for staff, as they are required to facilitate multiple PBL groups simultaneously.

#### Lack of training and experience in PBL

Many tutors come from medical schools that follow conventional teaching and learning methodologies, where PBL is not commonly used. *“There is no adequate PBL training for newly engaged staff; many of us are asked to facilitate PBL sessions without taking introductory training on how to lead PBL sessions. Without proper training, it is challenging to familiarize ourselves with the principles of PBL and develop the necessary skills and become effective facilitators.'* Furthermore, most of the tutors have served less than a year in their role as PBL tutors in the school, further intensifying their lack of experience in this teaching methodology.

#### Clinical-focused tutors in PBL

Students complained that most tutors, who are primarily clinical staff, tend to prioritize the clinical aspects of case scenarios rather than guiding students to delve into the underlying basic science. Students have expressed their dissatisfaction, stating that “*we are often asked clinical questions such as how to diagnose and manage a given patient presented in the case scenario, rather than being encouraged to discuss the fundamental science behind it”.* This mismatch between the tutors’ focus on clinical aspects and the students’ expectation to explore basic science concepts has led to frustration among the students.

#### Insufficient tutorial skills and evaluation-focused tutors

Some tutors exhibit inadequate tutorial skills, as they prioritize their own input and fail to create an inclusive learning environment that encourages student participation. Students complained that *‘Instead of facilitating discussions and fostering collaborative learning, some tutors tend to dominate the session with their own perspectives and start acting as a lecturer in the class room’.* Furthermore, students have expressed frustration with tutors who focus primarily on evaluating their performance rather than supporting their learning journey.

### Theme 3: PBL Case-related barriers

#### Ambiguous case scenarios

The Problem Based Learning (PBL) cases listed in the curriculum are often unclear, causing confusion for both students and tutors. Tutors have expressed their concerns, stating that “*some of the cases lack clarity and do not provide a clear objective for discussion. In certain cases, PBL cases are exceptionally difficult to understand, lacking a clearly stated objective ‘.*

#### Allocation of time in PBL cases

The allocation of time for each problem-based learning (PBL) case is a frequent issue that has been highlighted by both students and tutors. A common comment shared by most students was that “giving equal time for all cases is not fair.” They further explained that “*The current practice of allocating equal time for each PBL case is problematic. Not all cases require the same amount of time for discussion and exploration. Some cases have more extensive underlying concepts that require additional time for in-depth discussion. On the contrary, other cases may be relatively straightforward and require less time for discussion.*

#### The timing of PBL delivery

One of the issues raised by students is the lack of a standard way to deliver problem-based learning cases. This includes the misalignment or poor arrangement of PBL cases with class lectures. The students said that “*We usually discuss cases in our PBL session before we learn the basic science lecture in the class; which is one of the reasons not to actively participate during the first session of the PBL.”* They suggested that it would be better if PBL was given after taking some class room lectures on the system and professional competency development (PCD), so that they could easily understand the case scenarios easily.

### Theme 4: assessment-related barriers

#### Subjectivity in assessment methods

Despite the existence of an assessment tool prepared by the department, many tutors do not utilize it. Instead, they rely on their subjective judgment to evaluate students. Students said that “*most tutors do not use the assessment check list; they simply evaluate us based on their subjective judgment and that creates disappointment for students”*.

On the contrary, the teachers have expressed their reservations about the assessment tool. Most tutors said that *‘the assessment tool is too complex and subjective to apply; it is tedious and time consuming to use it.’*

#### Lack of ongoing evaluation

Students complained that *‘Many tutors evaluate us at the end of the module or occasionally during the course, rather than providing continuous assessment and feedback.’* This practice has led to dissatisfaction among students who expressed their disappointment as ‘it *is unfair and does not accurately reflect our efforts and capabilities’.* They added ‘someone *smart on the last day can get a good result, which is unfair; we have to be evaluated on an ongoing basis with appropriate positive feedbacks given to each student so that we can improve our weak side’.*

## Discussion

This study identified barriers to implementing PBL that can be classified into four main categories: student-related, tutor-related, case scenario-related, and assessment-related. Student-related barriers such as resistance for PBL implementation, work overload, lack of common understanding between students, PBL session dominated by few students, students over expectation from the tutor, and lack of good communication skills are the main student-related barriers identified in this research. Studies done in Saudi Arabia, Brazil, Ghana and Malaysia also showed similar factors influencing the implementation of PBL [7, 10, 17, 18].

Some students may resist the implementation of PBL because it requires a different approach to learning compared to traditional didactic methods. They may be adapted to passive learning styles and find the transition challenging. In some cases, a small number of students may dominate PBL sessions, either due to their assertiveness or their level of preparedness. This can lead to other students feeling marginalized or disengaged from the learning process.

Addressing these student-related barriers requires a multifaceted approach, including providing clear guidance and support for students transitioning to PBL, fostering a collaborative learning environment, and offering resources and training to develop essential communication and problem-solving skills. Furthermore, promoting a culture of openness to new learning methods and addressing concerns about workload can help alleviate resistance to the implementation of PBL implementation among students [13, 19, 20].

The main barriers related to the tutor identified were lack of motivation, lack of experience and training, tutors with clinical orientation, and tutors focused primarily on evaluation. Similar findings were identified in studies conducted in Ethiopia, Azerbaijan, Indonesia, Pakistan and Korea [21–25]. This is likely due to the fact that many tutors graduated from traditional teacher-centered curricula and lack the experience and skills needed to effectively facilitate and evaluate problem-based learning (PBL). Therefore, it is essential that the tutor training be continuous and comprehensive, allowing the tutor to internalize and implement PBL methods effectively.

Case-scenario-related barriers were the other thematic area assessed in this research. In this regard, unclear case scenarios, not enough time allocated, and lack of standard way of case delivery system were the most important points mentioned by the study participants. The objectives and scope of some PBL sessions are not clear and the time given for each session is not proportional to the actual time needed to accomplish the session. Some case scenarios lack quality and do not stimulate critical thinking and self-directed learning. Learning objectives also lack integrity of different disciplines and do not promote collaborative learning. Studies in Saudi Arabia, Ghana, and Egypt also revealed similar findings [7, 26, 27].

Using problem-based learning (PBL) as a teaching methodology can be highly effective, especially when PBL cases are standardized and accompanied by clearly defined objectives and scopes. This approach not only ensures consistency, but also reduces the workload of students by eliminating confusion about which materials to study for their preparation. By providing structured PBL cases with clear learning objectives and delineated scopes, educators can streamline the learning process and improve the overall effectiveness of PBL sessions [20].

Another assessment-related barrier highlighted by the students in this study was the subjectivity of assessment methods. In addition, students expressed concerns about the use of one-time spot evaluations, which they perceived as problematic. Studies done in Ethiopia and Ghana also showed similar findings [7, 28]. Although there is an assessment tool prepared by the department, it lacks objectivity and is exposed for biased assessment of students, and that has to be clarified and objective. Tutors should provide progressive evaluation and feedback that would allow students to determine whether they reached requirements, went off track from the objectives, and reflection on processes of learning [29].

### Limitation of the study

The study was carried out in a single medical school, and therefore the findings may not accurately reflect the comprehensive national barriers associated with the implementation of problem-based learning and large-scale nationwide studies are recommended.

## Conclusions and recommendations

Lack of both tutor and student commitment, lack of standardized cases, absence of a recognition of staff input, gap in communication skills, work overload, lack of continuous training and at the time of evaluation of students were identified as the main barriers to the implementation of PBL. These findings imply that continuous tutor training in PBL case facilitation, assessment, and evaluation is crucial. Standardized PBL cases should be prepared ahead and stored in the PBL case bank to ensure a continuous supply of varied cases for effective PBL facilitation.

## Data Availability

The data sets used and/or analyzed during the current study are available from the corresponding author on reasonable request.

## Abbreviations

DBU: Debre Berhan University
FGD: Focus Group Discussion
KPI: Key Performance Indicator
PBL: Problem-Based Learning
WHO: World Health Organization
